# Clinical Lecture

**Published:** 1861-10

**Authors:** 


### CLINICAL LECTURE.

Extract from a Clinical Lecture on Diseases of Women, by Augustus K. Gardner, M. D., Professor of Clinical Midwifery and Diseases of Women. Reported in the *American Medical Monthly:*

"Coition is so important a Physiological function, and it is so difficult for the young practitioner to learn respecting the ills which arise from its abuse, either from actual experience of their treatment, or by their study in scientific works which allude but very imperfectly to them, that the theme may well deserve your serious and most studious attention, as it may not only save embarrassment, but also enable you the more easily to recognize and properly treat the many ills which result from over action in this direction. The earliest period to which the physician is summoned on account of physical injury to the female, is immediately after marriage. It is too often the case that, inspired with the idea that every man must act manfully his part, that the husband does it brutally, and the immediate result is a *laceration of the hymen fourchette* and soft parts, followed by hemorrhage some times so profuse and uncontrollable as to demand the physician's attendance. Some times, instead of any laceration, with rupture of any vessel, the parts are much bruised, with *ecchymosis and extravisation with the labia majora* which become swoolen and fainful."

The effect of coitus is very frequently seen in the *inflammation of the labia-vaginal gland,* sometimes called Hugier's gland from its recent discover and pathologist. *Vaginitis* is also the direct result of excessive coition. This complaint does not materially differ from gonorrhœa, unless it be that the latter is more virulent. Indeed I am very doubtful if gonorrhœa should be considered a specific disease; certainly I have seen cases of vaginitis not different in any respect from gonorrhœa after a connection of unquestionable purity; and it is generally recognized that urethritis in the male is often produced without an impure connection, being the result of uterine leucorrhœa of an acrid character. Vaginitis is unquestionably the product of excessive sexual intercourse, and we find it perhaps more frequently in the wives of young sanguine clergymen, and other men of like continent habits; unlike the majority of the young men of cities, at least at the present day, their youth is spent in abstinence and imagination. Marriage with them is a right which justifies the freest and fullest indulgence of their pent-up passions, and the consequence is that they are apt to go to such excess that disease is the result. Coitus does not, in the normal condition of the uterus, produce disease of the surface of the cervix. It, however, if very frequent, brutal, or in consequence of a too long virile organ, will produce congestion of the organ; then, from its inflamed condition, abrasion of the mucus membrane; from a consequent hyperæmia, hypertrophy, and increased weight, consequent prolapsus, and a whole train of depending symptoms. Where these symptoms exist, if acute disease continues, coitus is inconsistent with cure. When, however, we have chronic hypertrophy of the cervix, endometritis, as in the case now before us, I find that moderately frequent intercourse, is beneficial, and so far from being the cause of injury, acts as a direct stimulus to the parts, unloading the congested and turgid glands, and by exciting them to a more profuse secretion, tends to reduce the hyperæmia of the organ. Where hœmorrhage follows immediately after coition, with or without pain, there is always some disease present. It does not generally proceed from ulcerations, unless they be of the *fungous variety,* but it is usually an evidence of more serious disease, perhaps of a small mucus polypus, or a fibroid, either pediculated or sub-mucus, or of a cancerous or phagædenic ulcer. The hœmorrhage produced by coition from a mucus polypus not larger than a pea, is sometimes exceedingly persistent and debilitating.

In the pregnant, coition is often to be entirely prohibited, especially in those who have had frequent abortions, until the usual period for abortion has been assuredly passed, and resumed only with the greatest care. In fact, excessive intercourse will of itself produce abortion, as certainly and for the same reason as the water douche, by simple uterine irritation. This is, in my opinion, the reason why prostitutes so rarely become mothers.

